# CTE-type tau filaments in Alzheimer’s disease with co-morbid LATE-NC

**DOI:** 10.1007/s00401-026-03052-z

**Published:** 2026-07-07

**Authors:** Jaimin K. Rana, Emile S. Pinarbasi, Martin G. Fernandez, Vikas Navratna, Kyle S. Conway, Andrew P. Lieberman, Sami J. Barmada, Shyamal Mosalaganti

**Affiliations:** 1 Life Sciences Institute, University of Michigan, Ann Arbor, MI 48109 USA; 2 Program in Chemical Biology, University of Michigan, Ann Arbor, MI 48109 USA; 3 Department of Pathology, University of Michigan, Ann Arbor, MI 48109 USA; 4 Department of Biophysics, College of Literature, Science, and the Arts, University of Michigan, Ann Arbor, MI 48109 USA; 5 Department of Neurology, University of Michigan, Ann Arbor, MI 48109 USA; 6 Department of Biological Chemistry, University of Michigan, Ann Arbor, MI 48109 USA; 7 Department of Cell and Developmental Biology, University of Michigan, Ann Arbor, MI 48109 USA

Alzheimer's disease neuropathologic change (ADNC) is the most common neurodegenerative pathology, characterized by extracellular amyloid-β (Aβ) plaques and intracellular neurofibrillary tangles composed of tau [2]. Cryo-electron microscopy (cryo-EM) analysis of patient-derived tau filaments in ADNC revealed two different structural polymorphs: paired helical filament (PHF) or straight filament (SF), where two tau protofilaments adopt a ‘C’ shape and form an interface mediated by amino acids 332–336 (PHF) or 317–324 and 312–321 (SF). The structural signatures of PHF and SF polymorphs—collectively referred to as the AD-fold—are highly conserved across sporadic and familial ADNC [5, 7, 24, 25], and are also found in primary age-related tauopathy (PART) [24]. To date, structural characterization of pathological aggregates in ADNC has been restricted to “pure ADNC,” despite the high prevalence of co-morbid pathologies [10, 19, 21]. In particular, up to 50% of patients with ADNC also have limbic-predominant age-related TDP-43 encephalopathy neuropathologic change (LATE-NC) [3, 17], defined as mislocalized and aggregated TDP-43 in medial temporal structures, including the amygdala and hippocampus. Notably, there is evidence of synergy between ADNC and LATE-NC; patients with co-morbid LATE-NC (AD + LATE-NC) have a higher Braak stage and a severe clinical decline [8, 9, 11, 16, 18]. However, whether co-aggregating proteins such as TDP-43 can influence tau structure remains unknown. Here, we show that in two AD + LATE-NC cases, tau adopts the chronic traumatic encephalopathy (CTE) fold in addition to the classical AD-fold.

Two cases with high ADNC (Braak 5, Thal 5), co-morbid LATE-NC (stage 2 or 3), and age-related tau astrogliopathy (ARTAG) were selected for analysis (Tables S1–3). Phosphorylated tau immunostaining in the amygdala of two AD + LATE-NC cases revealed dense tau pathology, predominantly comprising NFTs and neuropil threads. Aβ immunostaining demonstrated frequent diffuse and neuritic amyloid plaques (Fig. 1a, b, left and right panels, respectively). Phosphorylated TDP-43 (pTDP-43) immunostaining demonstrated dense pathology in the amygdala (Fig. 1a, b, middle panel). To explore the molecular content of these inclusions, we extracted sarkosyl-insoluble material that revealed enrichment of high-molecular weight species of pTau, Aβ, and pTDP-43 in these samples, as verified by immunoblotting (Fig. S1a) and immunoelectron microscopy (immuno-EM; Fig. S1b). Consistent with the accumulation of TMEM106B in age-related diseases [4, 23], we also observed TMEM106B fibrils in detergent-insoluble material from AD + LATE-NC (Fig. S1b).

**Fig. 1:**
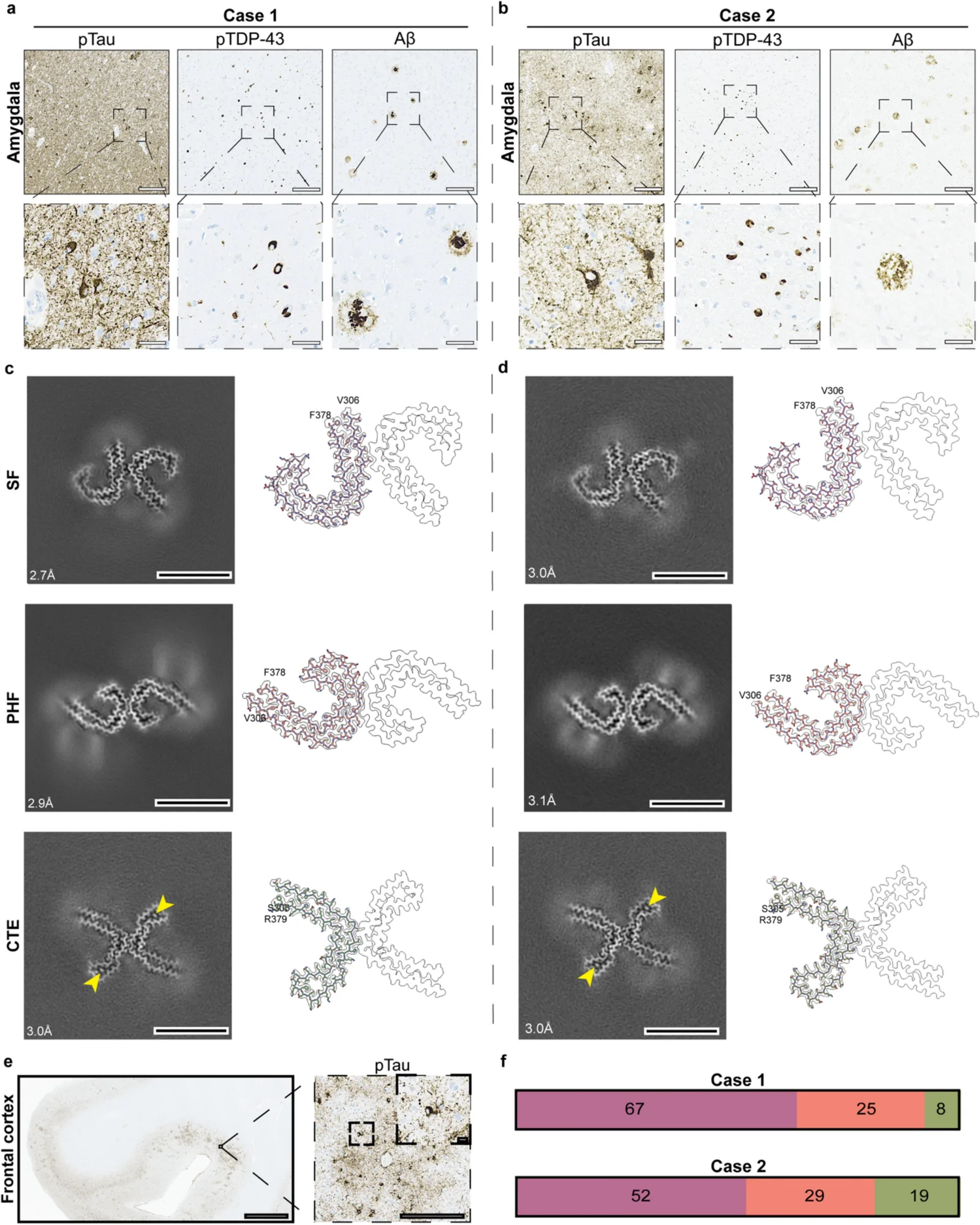
CTE-fold tau filaments are present in AD co-morbid with LATE-NC. **a**, **b** Representative images of amygdala from case 1 (**a**) and case 2 (**b**) immunostained with AT8 (specific for pS202 and T205 tau; pTau, top left), anti-pS409/S410 TDP-43 antibody (pTDP-43, middle), and anti-amyloid-ß antibody (Aβ, top right). Insets (bottom) show high magnification views. Scale bars: 300 µm (top) and 60 µm (bottom). **c**, **d** Structures of sarkosyl-insoluble tau filaments isolated from the amygdala of case 1 (**c**) and case 2 (**d**). Cross-sections through the cryo-EM reconstructions, perpendicular to the helical axis (left), and the cryo-EM density map with a fit of the tau atomic model (right) of SF (top), PHF (middle), and CTE (bottom). The cross-sections are approximately one rung thick. Resolutions of structures were calculated from the gold-standard Fourier shell correlation between two half-maps. Yellow arrowheads highlight non-proteinaceous density at the center of the β-helix turn in CTE-fold tau. Scale bar: 10 nm. Right panels: cryo-EM cross-sectional densities for two identical protofilaments fitted to tau amino acids F378 to V306 for SF (PDB: 5O3T) and PHF (PDB: 5O3L), and R379 to S305 for CTE (PDB: 6NWP). **e** AT8 immunohistochemistry of the frontal cortex, showing pTau distribution. *Indicates depth of the sulcus. The lumen of the vessel is outlined in red. Scale bar: 3 mm (left) and 200 µm (insets). **f** Stacked bar graph depicting the percentage of tau structural polymorphs in AD + LATE cases 1 and 2, as determined by cryo-EM analysis

Cryo-EM analysis on the sarkosyl-insoluble material (Fig. S2**a**) allowed us to determine the structures of tau SF at 2.7 and 3.0 Å and PHF at 2.9 and 3.1 Å, for cases 1 and 2, respectively (Fig. 1c, d**,** Figs. S2**b and 3; Table S4**). De novo model building and fitting of the previously published SF and PHF tau structures, followed by real-space refinement, yielded nearly identical models (mean RMSD < 1 Å) (Fig. S2**c, d**). Our structures showed no deviation from previously reported tau structures in AD patients [5, 7], providing additional support for the conserved signature of AD-fold tau even in mixed pathologies such as AD + LATE-NC.

Besides AD-fold tau, we observed distinct class averages resembling tau conformations previously identified in CTE [6] in the initial reference-free 2D classification (Fig. S2b). Notably, this tau conformation had not previously been reported in cases of ADNC [5, 7, 24]. We resolved the structure of CTE-fold tau at a resolution of 3.0 Å in both cases (Fig. 1c, Fig. S2b, and Fig. S3; Table S3), which is formed by two protofilaments adopting a more open β-helix turn conformation in comparison to PHF/SF tau [6]. We were able to unambiguously fit the previously published model for CTE-type I, consisting of two distinct protofilaments, each composed of core residues spanning S304 to R379, into our structure. Although prior investigations of clinically diagnosed CTE cases highlighted two types of tau filament morphologies (types I and II), we were unable to identify type II filaments in our AD + LATE-NC cases. This is consistent with the predominance of type I over type II fibers in CTE, potentially owing to the relatively weak interaction at the type II protomer interface [6].

CTE neuropathologic change (CTE-NC) is a pattern of tau pathology that is tightly associated with repetitive head injury, particularly in the context of contact sports [13–15]. Current consensus criteria require at least one cortical CTE lesion, defined as neuronal tau aggregates, with or without astrocytic tau, arranged around small vessels in the depths of cortical sulci [1]. Based on our structural findings, we performed additional cortical sampling per consensus guidelines [1]. This revealed a single CTE lesion in case 1 (Fig. 1e, Figs. S4 and 5), indicating previously unrecognized CTE-NC. However, despite extensive sampling (22 additional sections), we did not find any lesions in case 2 meeting all consensus criteria (Fig. S6). Notably, a comprehensive review of both patients’ clinical records and obituaries did not reveal any history of traumatic head injury or contact sport participation.

Co-morbid neuropathologies are common, found in up to 80% of neurodegenerative disease post-mortem samples [12, 22]. However, most structural studies only examine “pure” cases that exhibit a single neuropathology. Our cryo-EM analysis of two cases of AD + LATE-NC uncovered CTE-fold tau in addition to the expected AD-fold tau, which was associated with CTE-NC in one case (Fig. 1f). Our study highlights some of the diagnostic challenges associated with CTE-NC. CTE-NC is rarely found in patients without a known history of repetitive head injury [20], and its pathognomonic cortical lesions may be sparse and easily missed despite extensive sampling [1]. In addition, extracortical CTE-NC tau pathology can be difficult to distinguish from ADNC [1]. Consistent with this, CTE-NC was not suspected on initial review; instead, diagnostic lesions were identified only upon additional sampling prompted by our structural analyses.

Our study also suggests that tau structure may not always correlate with neuropathology. One case contained CTE-fold tau but did not meet criteria for CTE-NC despite extensive sampling. Both cases had ARTAG, yet the previously described structural correlate of ARTAG, AGD-fold tau [25], was absent. Together, these findings suggest that the relationship between tau conformation and neuropathology may vary by brain region or be influenced by co-morbid pathologies such as LATE-NC.

## Supplementary Information

Below is the link to the electronic supplementary material.

Supplementary file1 (PDF 2053 KB)

Supplementary file2 (DOCX 23796 KB)

## Data Availability

Cryo-EM maps of tau SF, PHF, and CTE have been deposited in the Electron Microscopy Data Bank (EMDB) under accession codes EMD-73448, 73449, 73450 for case 1, and EMD-73451, 73452, 73453 for case 2. All other materials used and data reported in the study are available upon request.
