# Correction: CTE-type tau filaments in Alzheimer’s disease with co-morbid LATE-NC

**DOI:** 10.1007/s00401-026-03069-4

**Published:** 2026-08-20

**Authors:** Jaimin K. Rana, Emile S. Pinarbasi, Martin G. Fernandez, Vikas Navratna, Kyle S. Conway, Andrew P. Lieberman, Sami J. Barmada, Shyamal Mosalaganti

**Affiliations:** 1 Life Sciences Institute, University of Michigan, Ann Arbor, MI 48109 USA; 2 Program in Chemical Biology, University of Michigan, Ann Arbor, MI 48109 USA; 3 Department of Pathology, University of Michigan, Ann Arbor, MI 48109 USA; 4 Department of Biophysics, College of Literature, Science, and the Arts, University of Michigan, Ann Arbor, MI 48109 USA; 5 Department of Neurology, University of Michigan, Ann Arbor, MI 48109 USA; 6 Department of Biological Chemistry, University of Michigan, Ann Arbor, MI 48109 USA; 7 Department of Cell and Developmental Biology, University of Michigan, Ann Arbor, MI 48109 USA

**Correction: Acta Neuropathologica (2026) 152:3** 10.1007/s00401-026-03052-z

In this article, five supplementary tables were inadvertently published in the main article. They have now been removed from the original published article.

The original article has been corrected.

