# Supplementary material for: CTE-type tau filaments in Alzheimer’s disease with co-morbid LATE-NC
Source: Acta Neuropathol. 2026 Jul 7;152(1):3. doi: 10.1007/s00401-026-03052-z (PMC13337903; doi:10.1007/s00401-026-03052-z)
Supplement: Supplementary file 1 — Supplementary file1 (PDF 2053 KB) [file 401_2026_3052_MOESM1_ESM.pdf]

Supplementary Information for:

CTE-Type Tau Filaments in Alzheimer's Disease with Co-morbid LATE-NC

Jaimin K. Rana[1,2,#], Emile S. Pinarbasi[3,#], Martin G. Fernandez[1,4], Vikas Navratna[1], Kyle S. Conway[3], Andrew P. Lieberman[3], Sami J. Barmada[5,*], and Shyamal Mosalaganti[1,2,4,6,7,*]

*Correspondence: sbarmada@med.umich.edu (S.J.B.); mosalaga@umich.edu (S.M.)

This file contains:

Methods

Supplementary figures 1-6

Supplementary Tables 1-5

References

Methods

Selection of cases

Cases of LATE in the University of Michigan brain bank were screened by a neuropathologist for the following: i) Dense TDP-43 pathology as assessed by immunohistochemistry for phospho-TDP43. ii) Absence of Lewy body pathology, or other co-morbid pathologies besides ADNC and ARTAG. iii) Availability of frozen tissue for structural analysis. This resulted in five cases. Of these five cases, only three had sarkosyl-insoluble fibrils morphologically compatible with TDP-43 by negative stain-EM; we therefore proceeded with single-particle cryo-EM on two of these cases. Because Alzheimer's Disease is associated with a wide spectrum of clinical features, particularly in the context of mixed pathologies, no emphasis was placed on the clinical presentation.

All brain tissues studied were obtained through the Michigan Brain Bank.

Clinical history and neuropathology

Individual 1 (Case 1) presented at age 82 with "leaning attacks" interpreted as transient ischemic attacks. At age 89, the individual began to show multi-domain cognitive impairment with difficulty navigating familiar places, word-finding difficulties, and trouble consistently taking medications. Cognition and motor function steadily declined until death at age 94. The clinical diagnosis, at death, was "mixed cognitive and motor disease".

Individual 2 (Case 2) presented at age 69 with memory impairment and was given a clinical diagnosis of Alzheimer's Disease. Cognition was relatively stable for the first few years, but then progressively declined until the individual's death at age 80.

Neuropathologic sampling and diagnoses were in accordance with the most recent consensus criteria [3, 4, 13, 14, 19].

Extended clinical and pathological information for all individuals is presented in Table S1.

Ethical Review and IRB approval

Because all brain tissue was obtained post-mortem through the Michigan Brain Bank, this study was exempt from IRB approval.

Immunohistochemistry

Detailed immunohistochemical analysis of different brain regions is provided in Tables S2 and S3 for cases 1 and 2, respectively. Immunohistochemistry was performed on a Ventana Benchmark Ultra immunostainer (Tucson, AZ). Slides were dewaxed, rehydrated, and subjected to antigen retrieval (details vary by antibody; see Table S5). Slides were incubated with the primary antibody and detected using the indicated system, as noted in Table S5. Slides were then counterstained with hematoxylin and coverslipped.

Immunofluorescence

Slides were dewaxed (xylene, 10 min), treated with 100% EtOH for 7 min, then underwent antigen retrieval in 1× RNAScope Ag retrieval solution (ACD Biosciences 322000) at 100°C for 15 min, followed by room temperature water for 15 seconds and then 100% EtOH for 3 minutes. After drying, the tissue was permeabilized with 0.1% Triton-X for 15 min and blocked for 1 hour at room

temperature in a blocking solution (0.1% Triton X-100, 2% fetal calf serum (Sigma, #F4135), and 3% bovine serum albumin (BSA, Fisher BP9703-100) in PBS. Tissue was then incubated overnight with antibodies (see Supplemental Table 2 for additional details) in blocking buffer. Tissue was then washed 3× in PBS and incubated at room temperature with Alexa Fluor 488 donkey anti-mouse (Invitrogen A32766) and Alexa Fluor 647 goat anti-chicken (Invitrogen A32933). Tissue was washed 3× in PBS, incubated for 10 min in 1:1000 Hoechst 33258 dye (Invitrogen, #H3569), then washed 3× in PBS, and mounted in mounting media (Prolong Gold Invitrogen, #P36930). Slides were allowed to dry overnight before imaging on a Leica Stellaris equipped with 5× air and 40× oil objectives.

Purification of filaments from patient tissue

Sarkosyl-insoluble material was extracted from flash-frozen amygdala using protocols described previously [2]. Briefly, ~1.0 g of tissue was homogenized in 40 ml buffer A (10 mM Tris-HCl, pH 7.5, 0.8 M NaCl, 10% sucrose, and 1 mM EGTA) using a polytron homogenizer and solubilized in 2% final sarkosyl solution for 1 h at 37 °C. The homogenates were centrifuged at 27,000 *g* for 15 min, and the supernatant was collected for further centrifugation at 166,000 *g* for 30 min. The pellets were resuspended in 6 mL g^{-1} buffer A containing 1% sarkosyl and homogenized using a hand-held probe sonicator. The homogenates were diluted 4-fold with buffer A containing 1% sarkosyl and incubated for an additional 30 min at 37 °C. The solubilized suspension was first centrifuged at 17,000 *g* for 5 min at 25 °C, followed by centrifugation at 166,000 *g* for 30 min at 25 °C. The resulting pellets were resuspended in 1 ml g-1 buffer A containing 1% sarkosyl and incubated for 1 h at 37 °C. Final centrifugation was performed at 100,000 *g* for 30 min at 25 °C. The sarkosyl-insoluble pellet was resuspended in 30 µl of buffer B (20 mM Tris-HCl, pH 7.4, and 150 mM NaCl).

Immunoblotting analysis

The tissue lysate (T), supernatant post-sarkosyl-solubilization (S), and the sarkosyl-insoluble pellet (I) were resuspended in a final working solution of 1× Laemmli buffer (Bio-Rad, #161-0747), heated at 95 °C for 5 min, resolved on 4-15% Criterion TGX™ (Bio-Rad) gels at 200 V for 40 min, and transferred onto PVDF membrane using Trans-Blot Turbo Transfer System (Bio-Rad). The membranes were blocked in PBS containing 1% Bovine Serum Albumin and 0.2% Tween-20 for 1 h at 21 °C. The membranes were subsequently incubated with primary antibodies: phospho-TDP-43 (Ser 409/410, 1:1000; Proteintech, #22309-1-AP), phospho-tau (Ser202/Thr205, 1:1000; Invitrogen AT8), Aß (1:1000, Milipore Sigma, #A3981), and TMEM106B[12] (1:800) overnight at 4 °C. The blots were washed three times with PBS containing 0.2% Tween-20 and subsequently incubated with HRP-conjugated secondary antibody, Goat anti-Rabbit (BD Biosciences, #554021) or Goat anti-Mouse (Millipore Sigma, 12-349) for 1 h at 21 °C. Following this, the membranes were again washed three times with PBS containing 0.2% Tween-20 and developed using Clarity Western ECL substrate (Bio-Rad, #1705061) and images using Bio-Rad ChemiDoc Imaging System.

Immunoelectron microscopy (Immuno-EM)

300 mesh carbon-coated copper grids (Electron Microscopy Sciences, #CF300-CU-50) were glow-discharged for 60 s at 5 mA (PELCO easiGlow). Sarksosyl-insoluble filaments were further diluted 1:10 in buffer B (20 mM Tris-HCl, pH 7.4, and 150 mM NaCl), and 3 µl of the sample was

applied to the grids, incubated for 3 min, and subsequently the excess liquid was blotted from the grids using a Whatman 1 filter paper (Cytiva, #1001090). Next, blocking buffer [PBS, pH 7.4, 0.1% w/v cold water fish skin gelatin (Aurion, CFWS Gelatin)] was applied, and the grids incubated for 10 min. Subsequently, excess liquid was blotted again, and primary antibody (see immunoblotting analysis; 1:20 in blocking buffer) was applied to the grids and incubated for 1 h. Following blotting, grids were washed five times with a blocking buffer, and excess solution was removed in between each wash. 12 nm Colloidal Gold AffiniPure Goat Anti-Rabbit IgG (Jackson ImmunoResearch Laboratories, #111-205-144) diluted 1:4 in blocking buffer was applied to the grids and incubated for 1 h. Grids were washed five times with Milli-Q water, and excess liquid was blotted off between washes. Finally, grids were stained with 3 µL of 0.75% uranyl formate (Electron Microscopy Sciences, #16984-59-1) for 1 min, washed twice with Milli-Q water, and the excess solution blotted off. The grids were imaged using a Morgagni transmission electron microscope (Thermo Fisher Scientific) at an acceleration voltage of 100kV. The images were recorded with a Gatan Orius SC200 CCD camera with a resolution of 2.1 Å/pixel and Digital Micrograph software (Gatan Inc).

Cryo-EM Sample preparation and data-collection

Sarkosyl-insoluble pellets were diluted 1:4 in buffer B (20 mM Tris-HCl, pH 7.4, and 150 mM NaCl) and treated with 0.4 mg ml-1 pronase (Sigma) before plunge-freezing. 2.5 - 3 µl of samples were applied to glow-discharged (60 s at 15 mA, PELCO easiGlow) R1.2/1.3 UltrAuFoil Au 300 mesh grids (Quantifoil GmbH). Grids were blotted for 3.5 s at 18 °C and 100% humidity with blot force 5, then immediately frozen in liquid ethane using a Vitrobot (Mark IV, Thermo Fisher Scientific). Images were acquired using Titan Krios G4i operated at 300 kV equipped with a bioQuantum energy filter with a slit width of 20 eV and a Gatan K3 direct electron detector. Automated collection was performed at a nominal magnification of 105,000×, yielding a calibrated pixel size of 0.83 Å/pixel with SerialEM v 4.0 [18]. A total of 50 frames were collected during a 2 s exposure, with an accumulated dose of 50.4 e^{-} $Å^{-2}$, and a defocus range of -2.5 µm to -1 µm.

Helical Reconstruction

All image processing was performed using RELION 5.0.0 [5]. Movies were gain-corrected, aligned, dose-weighted, and summed using the motion-correction algorithm implemented in RELION-5.0.0 [20]. Motion-corrected micrographs were used to estimate the contrast transfer function using CTFFIND4 [16]. Helical reconstruction of tau filaments was performed as described previously [8, 11, 17]. Briefly, the filaments were manually picked and extracted as segments with a box size of 1024 pixels, then downsampled to 128 pixels. Two rounds of reference-free 2D classification were performed, and particles were selected based on the hierarchical clustering of the filaments using the FilamentTools program, resulting in a homogenous subset of the class averages for each filament type [10]. Particle numbers at this stage were used to calculate percentage ratios of the polymorphs depicted in Fig. 1f.

In each case, initial models were generated *de novo* using *relion_helix_inimodel2d.py* as previously described, to avoid any model bias. For initial model generation, a subset of particles for each filament type was re-extracted with a box size of 768 pixels, downsampled to 384, subjected to reference-free 2D classification, and used as inputs. The volumes obtained were rescaled and used as initial references for 3D auto-refinement, starting with 8× binned particles

(6.64 Å/pixel) and progressing to an unbinned final refinement (0.83 Å/pixel). Optimization of helical parameters was performed once the map resolution was at 4.5 Å or lower, and a clear separation among β-sheets was apparent. In both cases, we achieved resolutions of 3.2-3.6 Å for all the structures. Bayesian polishing was performed to further improve the resolution of each map[20]. In both cases, 3D classification was performed to identify the best-aligned segments, followed by CTF refinement to further improve map resolution. Final 3D reconstructions from all cases (a total of 6 maps) were sharpened using the post-processing method implemented in RELION, and global resolution was estimated from Fourier shell correlation at a cutoff of 0.143 between the two independently refined half-maps. Post-processed maps were symmetrized with helical symmetry parameters in real space using the RELION Helix Toolbox program.

Model building

We build *de novo* models using ModelAngelo [9]. RMSD between the published structures and ModelAngelo models was less than 1. We therefore used published Protein Data Bank (PDB) models of PHF (PDB ID: 5O3L), SF (PDB ID: 5O3T), and CTE (PDB ID: 6NWP) to fit into the corresponding maps. For SF, PHF, and CTE, the published models were fit into corresponding Coloumbe potential maps as rigid bodies in ChimeraX[15]. After removing hydrogen atoms in COOT [6], real-space refinement was performed using PHENIX [1]. ChimeraX was used to visualize the structures and make figures.

Supplementary figures (1-6):

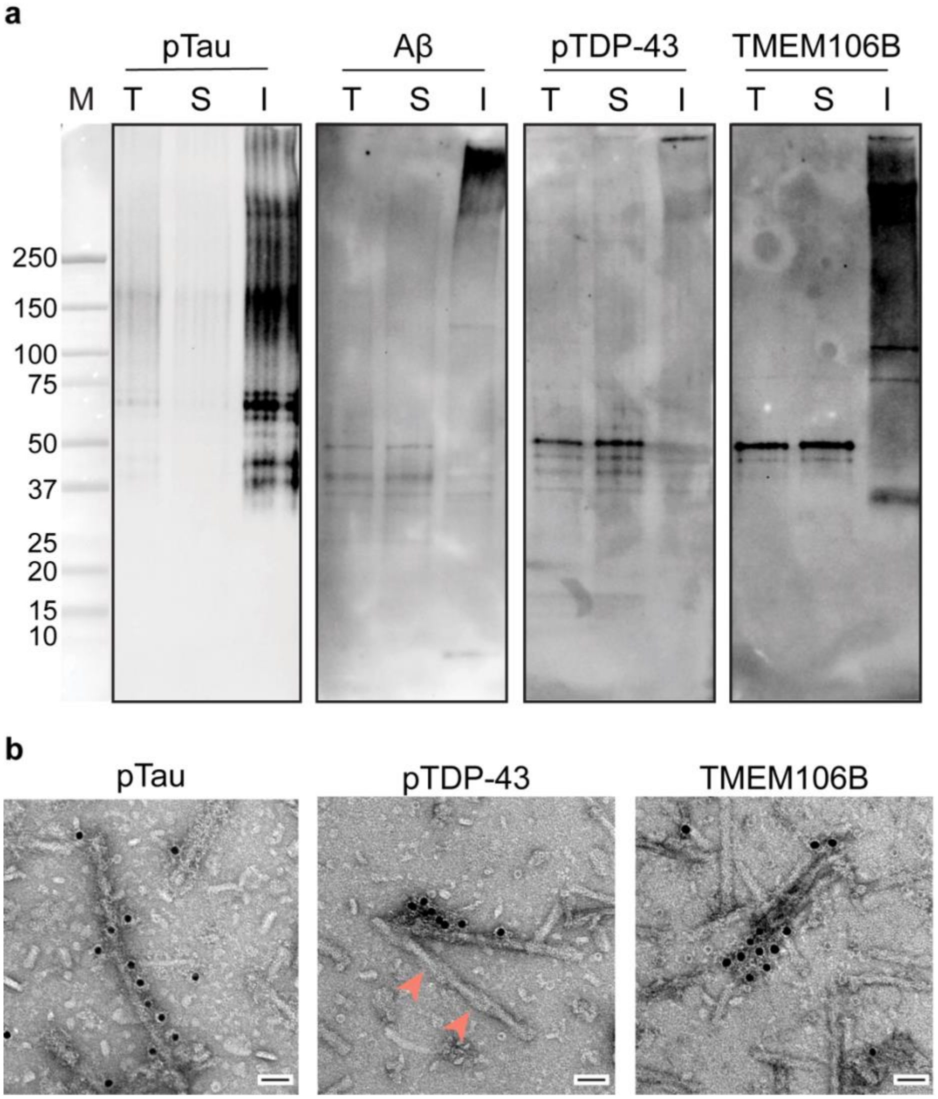

Fig. S1: Immunoblot analysis of insoluble inclusions from the amygdala of a patient with LATE with AD (Case 1). (a) Sarkosyl-insoluble material (I) was isolated from the amygdala, and probed with antibodies AT8 (specific for pS202 and T205 tau; pTau), anti-amyloid beta antibody (Aβ), anti-pS409/S410 TDP-43 antibody (pTDP-43), or anti-TMEM106B core [12]. Total lysate (T) and the soluble fraction (S) are also shown in each case. The Precision Plus Protein Dual Color (Bio-Rad) marker is denoted as M. (**b**) Representative negative stain EM images of immunogold-labeled sarkosyl-insoluble material (anti-pTau, left; anti-pTDP-43, middle; anti-TMEM106B, right) from Case 1. Arrowheads (warm pink, middle) point to a tau PHF fibril, as evidenced by the characteristic shape [7] that is not recognized by the anti-pTDP43 antibody, highlighting the selectivity of immunogold labeling. Scale bar: 50 nm.

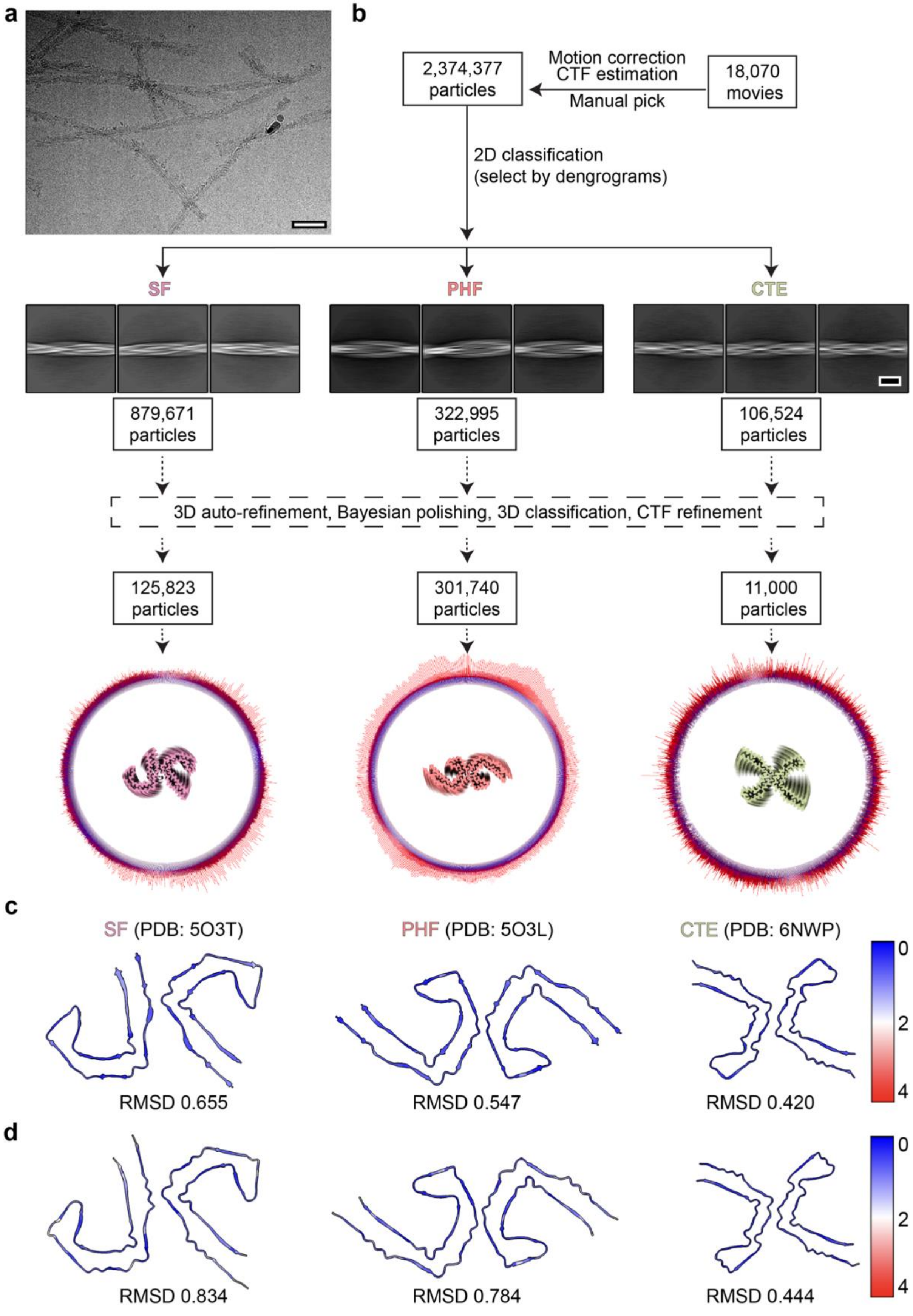
a
b
2,374,377 particles
Motion correction
CTF estimation
Manual pick
18,070 movies
2D classification
(select by dengrograms)
SF
PHF
CTE
879,671 particles
322,995 particles
106,524 particles
3D auto-refinement, Bayesian polishing, 3D classification, CTF refinement
125,823 particles
301,740 particles
11,000 particles
c
SF (PDB: 5O3T)
PHF (PDB: 5O3L)
CTE (PDB: 6NWP)
0
2
4
RMSD 0.655
RMSD 0.547
RMSD 0.420
d
0
2
4
RMSD 0.834
RMSD 0.784
RMSD 0.444

Fig. S2: Helical reconstruction workflow for tau filaments. a. Representative cryo-EM image of sarkosyl-insoluble material from amygdala. Scale bar, 50 nm. **b**. Data analysis workflow for the structure determination of tau filaments from case 1 as a representative example. ~18,000 movies were collected, motion corrected, and CTF was estimated on motion corrected micrographs using established procedures in Relion-5 [16, 20]. Fibrils were manually picked and sorted using dendrograms. The representative 2D classes are shown for SF, PHF, and CTE (scale bar, 10 nm). The filaments were separated, and all downstream steps were performed independently on each particle stack. **c**. Comparison between the model presented in the current study and the published model for SF (PDB: 5O3T), PHF (PDB: 5O3L), and CTE (PDB: 6NWP). The models are colored based on RMSD values: 0 (blue), 2 (white), and 4 (red). **d**. Comparison between the SF, PHF, and CTE models determined by ModelAngelo [9] and the published model for SF, PHF, and CTE. The models are colored based on RMSD values: 0 (blue), 2 (white), and 4 (red).

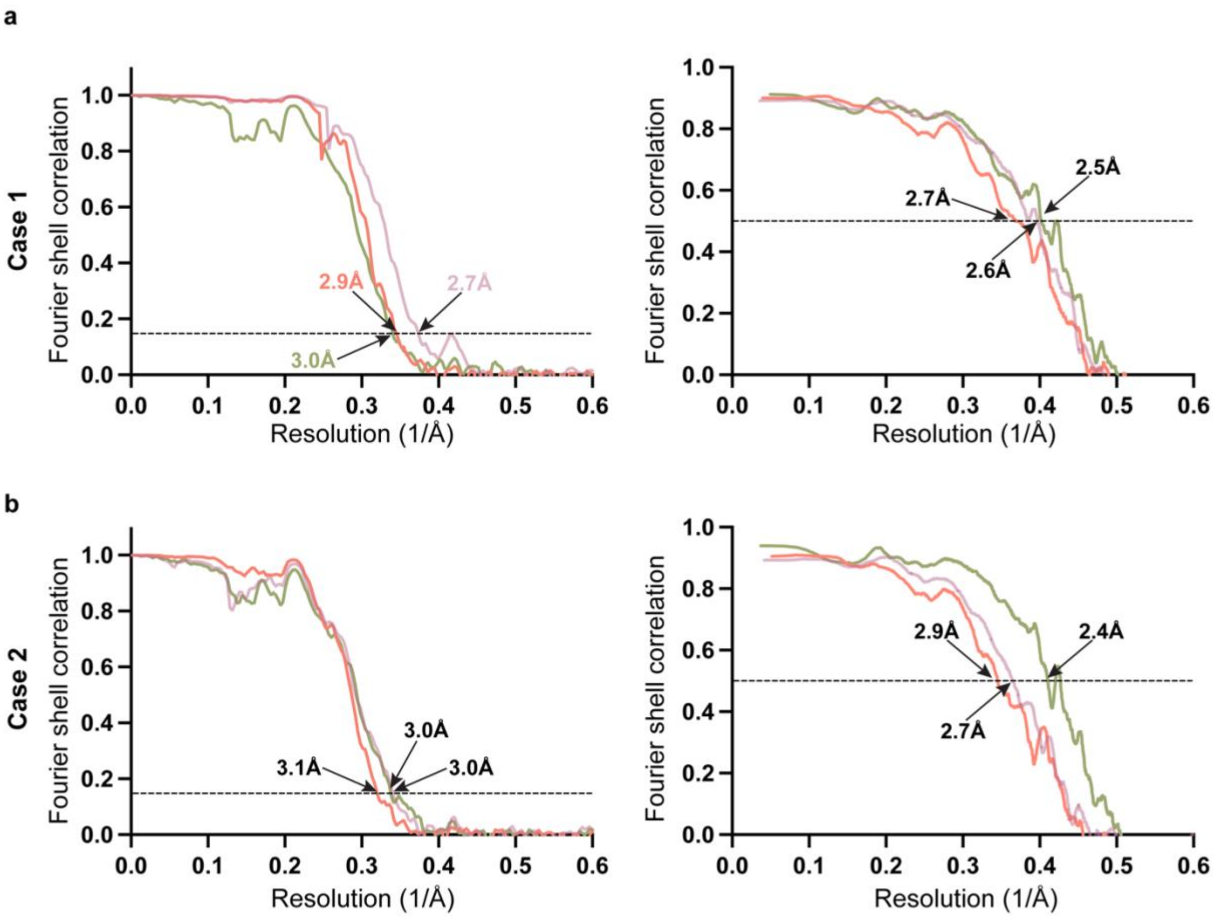

Fig. S3: Fourier shell correlation (FSC) curves. a-b. FSC curves of cryo-EM maps (left) and model to map validation (right) for LATE with AD cases (cases 1 and 2) analyzed in this manuscript. In each case, curves for SF, PHF, and CTE are colored in magenta, warm pink, and green, respectively. Resolution in angstroms is shown for the cryo-EM maps ($FSC_{0.143}$) and model to map ($FSC_{0.5}$).

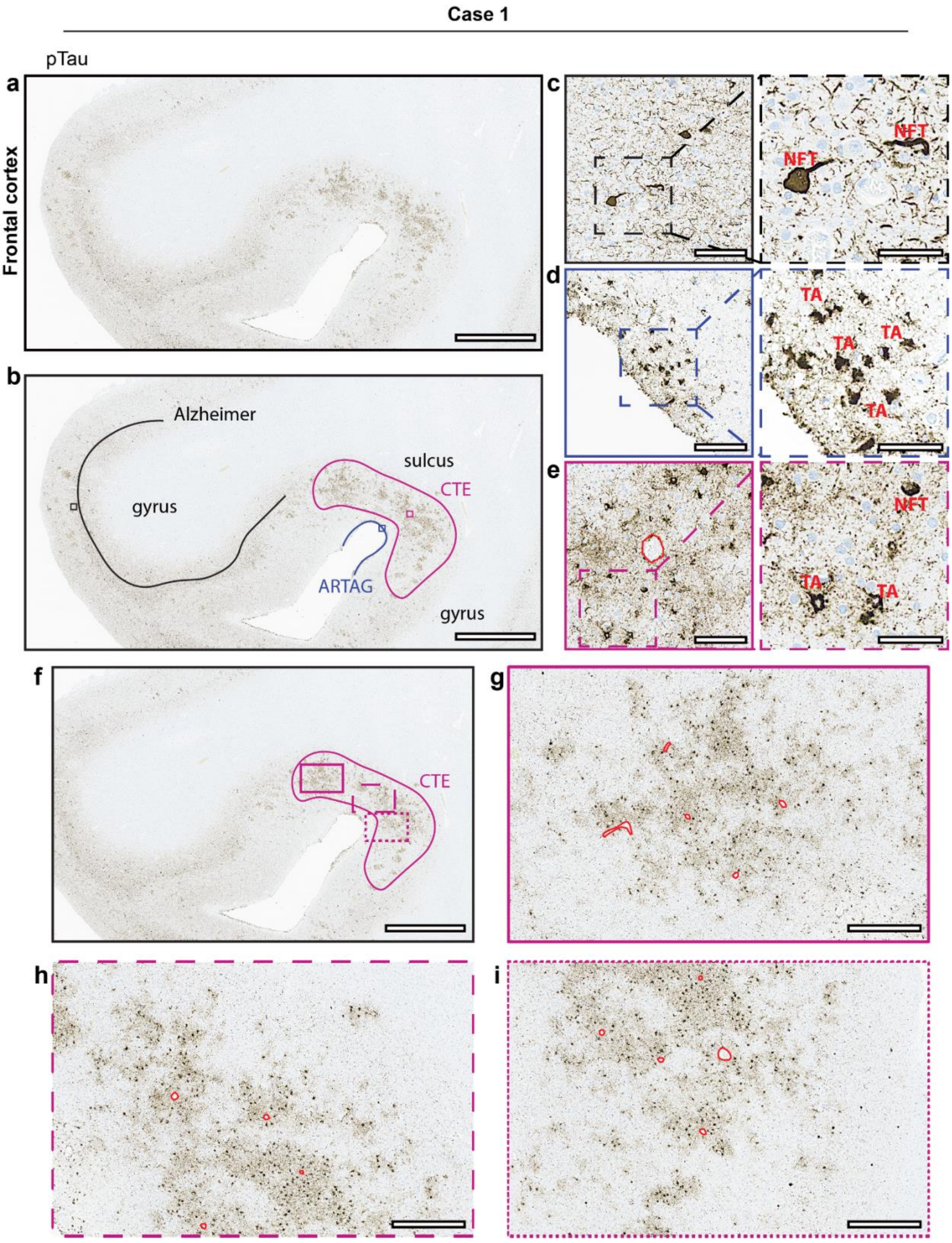
Case 1
pTau
Frontal cortex
a
b
Alzheimer
sulcus
gyrus
CTE
ARTAG
gyrus
c
NFT
NFT
d
TA
TA
TA
TA
e
NFT
TA
TA
f
CTE
g
h
i

Fig. S4: Annotation of diagnostic CTE features in Case 1. a. Unannotated image of the frontal cortex immunostained for AT8 (specific for pS202 and T205 tau; pTau). Scale bar, 3 mm. **b**. Same image as (a), with annotations of gyrus, sulcus, the Alzheimer's tau pathology (outlined in black), subpial ARTAG (blue), and CTE lesion (magenta). Boxes indicate the location of high magnification insets in c-e. Scale bar, 3 mm. **c-e**. Higher magnification insets of Alzheimer's pathology (c), ARTAG (d), and CTE (e). NFT = neurofibrillary tangle. TA = tufted astrocyte. Lumen of the blood vessel in (e) is annotated in red. Scale bars,100 μm (left) and 50 μm (right). **f**. Same image as (a) and (c), with solid, dashed, and dotted boxes designating the location of higher magnification inserts in (g-i). Scale bar, 3 mm. **g-i**. Higher magnification insets of solid (g), dashed (h), and dotted (i) regions. Lumens of blood vessels are outlined in red. Scale bar, 500 μm.

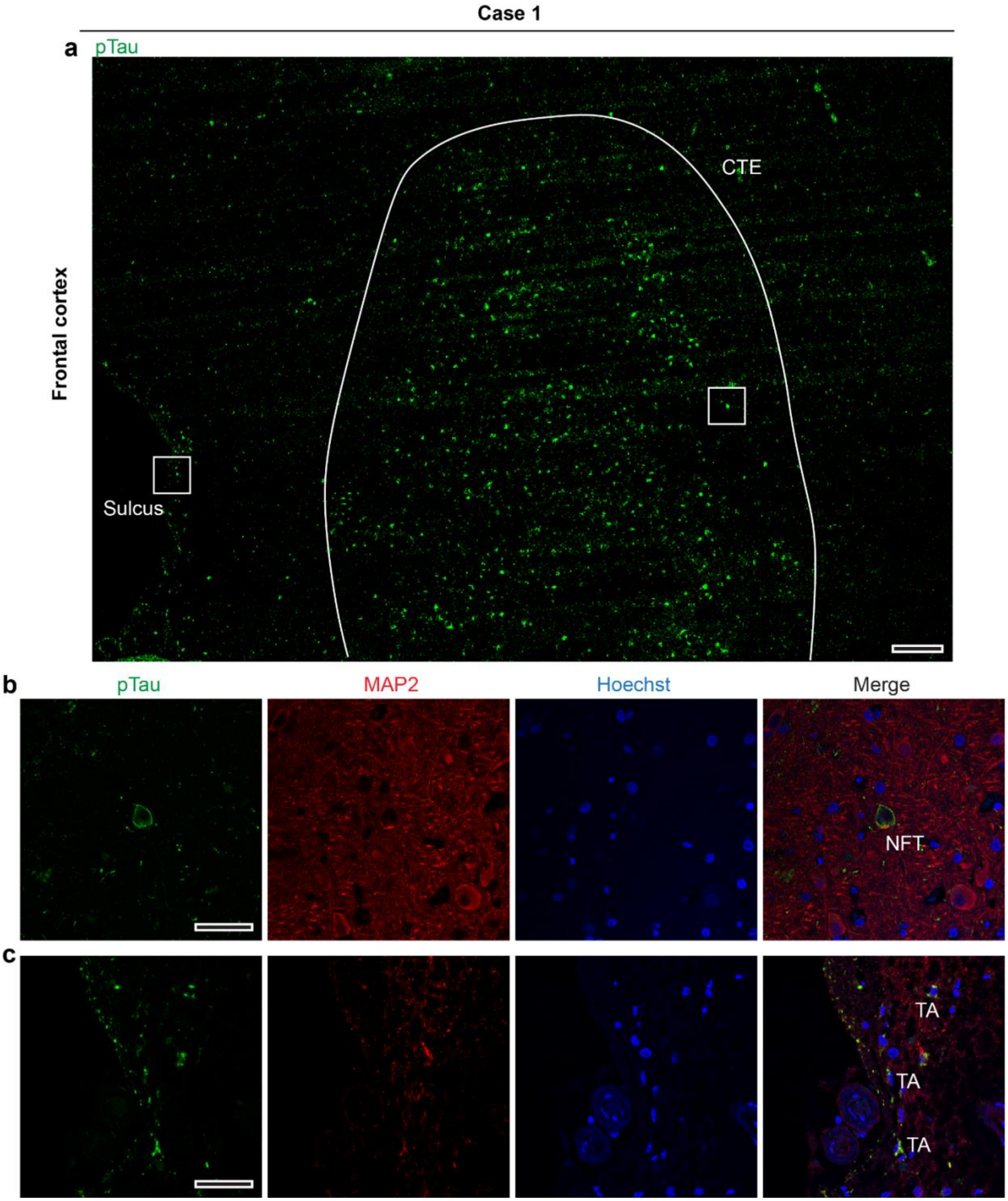

Fig. S5: Immunofluorescence of CTE lesion in Case 1. Immunofluorescence image of the frontal cortex stained with AT8 (specific for pS202 and T205 tau; pTau. Sulcus and CTE lesion annotated in white. (**b, c**). Insets showing higher-magnification images co-stained for MAP2 and Hoechst. NFT: neurofibrillary tangle. TA: thorny astrocyte. Scale bars 200 µm (a) and 50 µm (b, c).

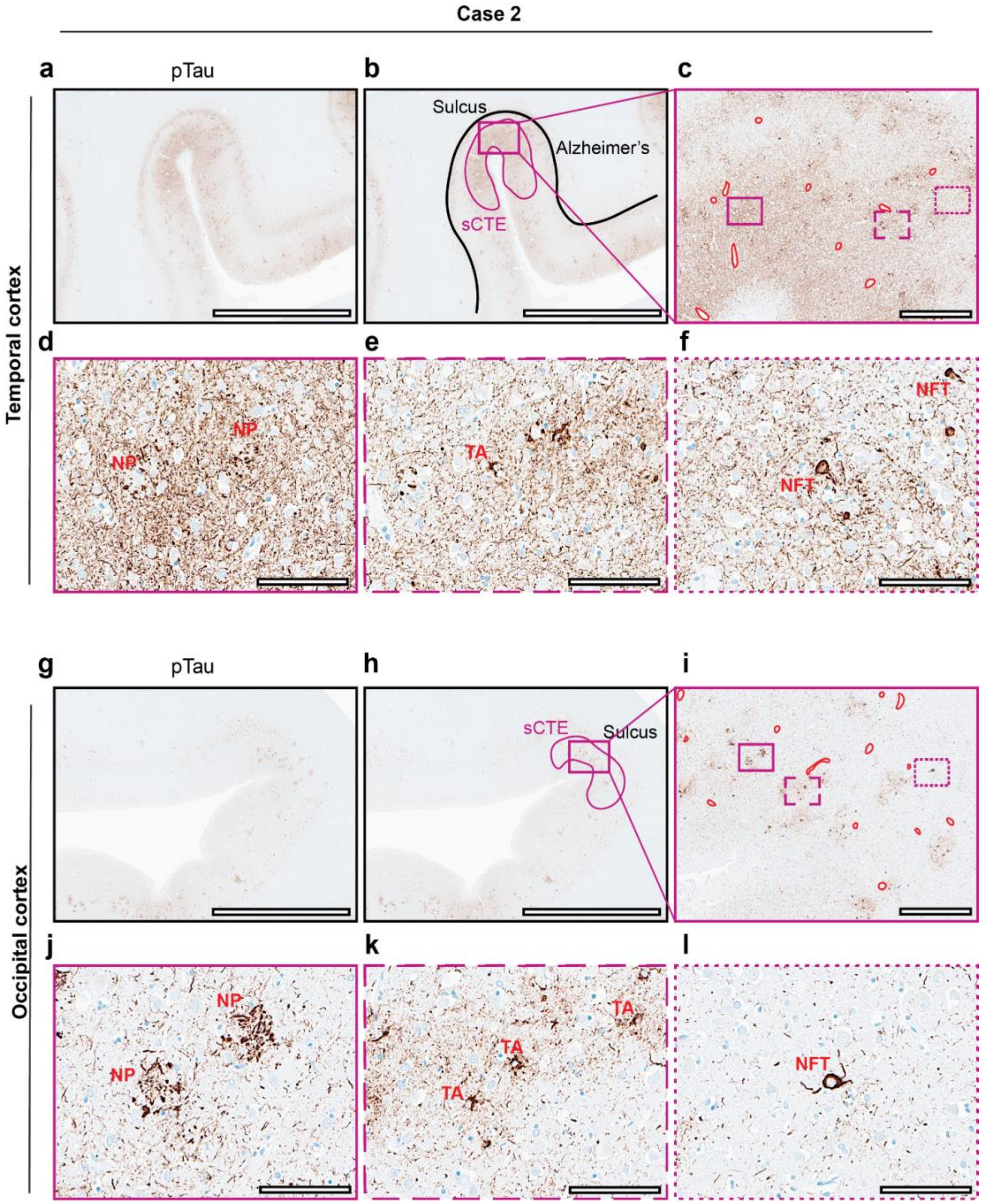

Fig. S6: Absence of definitive CTE lesions in Case 2. AT8 (specific for pS202 and T205 tau; pTau) immunostaining of suspicious areas in the temporal cortex (**a-f**) and the occipital cortex (**g-l**). Sulcus, Alzheimer's pathology (black), and focus suspicious for CTE (sCTE, magenta) are annotated in (b) and (h). Higher-magnification insets showing vessels outlined in red are shown in (c) and (i). Higher magnification insets shown in d-f and j-l. NP: neuritic plaque. TA: thorny astrocyte. NFT: neurofibrillary tangle. Scale bars 5 mm (a-b, g-h), 500 µm (c, i), and 100 µm (d-f, j-l).

Supplementary tables (1-5):

Table S1: Extended Clinical and Pathologic Information

	Age	Gender	Clinical Diagnosis	ADNC	Thal	Braak	CERAD	CAA	AS	VCING	LATE Stage	ARTAG	HS	CTE
**Case 1**	94	M	Mixed cognitive/motor disorder	High	5	5	2	Severe	Severe	Moderate	2	Yes, focal	No	Low
**Case 2**	80	M	Late onset AD	High	5	5	2	Severe	Severe	Moderate	3	Yes, widespread	Yes	None; features

AD: Alzheimer's Disease. ADNC: Alzheimer's Disease Neuropathologic Change. CAA: Cerebral Amyloid Angiopathy. AS: Arteriolosclerosis. VCING: Vascular Cognitive Impairment Neuropathology Guidelines. LATE: Limbic predominant age-related TDP-43 Encephalopathy. ARTAG: Age-related tau astrogliopathy. HS: Hippocampal Sclerosis. CTE: Chronic Traumatic Encephalopathy. Int: Intermediate

Table S2: Extended neuropathology for case 1

	pTau	β-amyloid	pTDP-43	α-synuclein
**Frontal cortex**	CTE lesion. Severe diffuse NFTs and neuropil staining. Focal ARTAG (subpial)	Diffuse and neuritic plaques, CAA	Rare neurite (depth of sulcus). No NCI	N/A
**Temporal cortex**	Severe diffuse NFTs and neuropil staining	Diffuse and neuritic plaques, CAA	Negative	N/A
**Parietal cortex**	Moderate diffuse NFTs and neuropil staining. Focal ARTAG (gray matter, subpial)	Diffuse and neuritic plaques, CAA	Negative	N/A
**Occipital cortex**	Cores of neuritic plaques, rare NFTs	Diffuse and neuritic plaques, CAA	Negative	N/A
**Additional cortical sections (5)**	NFTs and neuropil staining	N/A	Negative	N/A
**Anterior cingulate**	N/A	N/A	N/A	Negative
**Amygdala**	Severe diffuse NFTs and neuropil staining	Diffuse and neuritic plaques, CAA	Peripheral NCIs (NFT-like)	Negative
**Nucleus basalis and basal ganglia**	ARTAG (focal, subpial). NFTs in basal forebrain and striatum.	Diffuse plaques, CAA	Negative	N/A
**Hippocampus**	Dense neuropil staining and NFTs involving hippocampal formation, subiculum, parahippocampal gyrus, and temporal isocortex. ARTAG (subpial)	Diffuse and neuritic plaques, CAA	NCIs and neurites	N/A
**Thalamus and subthalamic**	Scattered NFTs and neuropil threads	Diffuse plaques	Negative	N/A
**Cerebellum**	Negative	Diffuse and neuritic plaques, CAA	N/A	N/A
**Midbrain**	N/A	Diffuse plaques, CAA	Negative	Negative
**Pons**	N/A	CAA	Negative	N/A
**Medulla**	N/A	Diffuse plaques, CAA	Negative	N/A
**Cervical spinal cord (2)**	N/A	N/A	Negative	N/A

NFT: Neurofibrillary tangle. ARTAG: Age-related tau astrogliopathy. CAA: Cerebral Amyloid Angiopathy.
NCI: neuronal cytoplasmic inclusion

Table S3: Extended neuropathology for case 2

	pTau	β-amyloid	pTDP-43	α-synuclein
**Frontal cortex**	Severe diffuse NFTs and neuropil staining. ARTAG (white matter, widespread)	Diffuse and neuritic plaques, CAA	NCIs and neurites (layers 2-3)	N/A
**Temporal cortex**	Severe diffuse NFTs and neuropil staining. ARTAG (gray matter, white matter, widespread). Accentuation of tau neuropil staining at depths of sulci	Diffuse and neuritic plaques, CAA	N/A	N/A
**Parietal cortex**	Moderate diffuse NFTs and neuropil staining, AD distribution. ARTAG (gray matter, white matter, widespread). Accentuation of tau neuropil staining at depths of sulci	Diffuse and neuritic plaques, CAA	N/A	N/A
**Occipital cortex**	Severe diffuse NFTs and neuropil staining, AD distribution. ARTAG (white matter, widespread)	Diffuse and neuritic plaques, CAA	N/A	N/A
**Additional cortical sections (17)**	Possible CTE lesion (Extended Data Fig. S6c). Accentuation of neuropil staining at sulcal depths (Extended Data Fig. S6d)	N/A	N/A	N/A
**Anterior cingulate**	N/A	N/A	N/A	Negative
**Amygdala**	Moderate NFTs and neuropil staining. Severe widespread ARTAG (subpial, white matter, perivascular)	Diffuse and neuritic plaques, CAA	Granular NCIs and short neurtes	N/A
**Nucleus basalis and basal gangli**	NFTs in basal forebrain and striatum. Subpial ARTAG (widespread)	Diffuse plaques	N/A	N/A
**Hippocampus**	Severe, widespread ARTAG (subpial, white matter, gray matter, subependymal) Dense neuropil staining and NFTs involving hippocampal formation, subiculum, parahippocampal gyrus, and temporal isocortex	Diffuse and neuritic plaques, CA	NCIs and neurites	N/A
**Thalamus and subthalamic**	NFTs and neuropil threads. Widespread ARTAG (subpial, subependymal)	Diffuse plaques	N/A	N/A
**Cerebellum**	Focal ARTAG (subependymal)	Diffuse and neuritic plaques, CAA	N/A	N/A
**Midbrain**	NFTs and neuropil threads. Subpial ARTAG	Diffuse plaques, CAA	N/A	Negative
**Pons**	NFTs and neuropil threads. Subpial ARTAG	Diffuse plaques	N/A	N/A
**Medulla**	Widespread ARTG (subpial, perivascular)	Diffuse plaques	N/A	N/A

NFT: Neurofibrillary tangle. ARTAG: Age-related tau astrogliopathy. CAA: Cerebral Amyloid Angiopathy. NCI: neuronal cytoplasmic inclusion

Table S4: Cryo-EM data collection and refinement statistics

	**Case 1**			**Case 2**		
**Data collection and processing**						
Microscope	Titan Krios			Titan Krios		
Voltage (kV)	300			300		
Detector	Gatan K3			Gatan K3		
Magnification	105,000			105,000		
Exposure ($e^-/Å^2$)	50			50		
Defocus range (μm)	-1 to -2.5			-1 to -2.5		
Pixel size (Å)	0.83			0.83		
**Refinement**	**SF EMD-73448**	**PHF EMD-73449**	**CTE EMD-73450**	**SF EMD-73451**	**PHF EMD-73452**	**CTE EMD-73453**
Box size	384	384	384	384	384	384
Symmetry imposed	C1	C1	C1	C1	C1	C1
Initial particle number	879,671	322,995	106,524	168,627	94,488	62,905
Final particle number	125,823	301,740	11,000	17,679	93,639	11,806
Map resolution (Å)	2.7	2.9	3.0	3.0	3.1	3.0
FSC threshold	0.143	0.143	0.143	0.143	0.143	0.143
Helical rise (Å)	4.78	2.39	2.38	4.78	2.38	2.38
Helical twist (°)	-1.07	179.46	179.41	-1.07	179.47	179.41
Map sharpening B-factor ($Å^2$)	-44.66	-73.81	-38.24	-30.14	-89.64	-48.57

Table S5: Antibodies used in this study

Immunohistochemistry

Antibody	Clone	Species	Quality Status	Vendor	Catalog #	Detection	Retrieval	Incubation	Dilution
pTau	AT8	Mouse	IVD	Thermo Fisher	MN1020	Ultraview	CC1 36 min	32 min at 36°C	1:200
ß-Amyloid	6F/3D	Mouse	IVD	Dako	M087201-2	Optiview	Formic Acid-60 min	92 min No heat	1:50
pTDP-43	1D3	Rat	IVD	BioLegend	829901	Discovery OmniMap anti-Rat HRP	Discovery CC1 for 32 min	32 min at 32 °C	1:500
α-synuclein	LB509	Mouse	IVD	Thermo	180215	Ultraview	Prot 2-16 min	32 min at 37 °C	1:100

Immunoblotting

Antibody	Clone	Species	Vendor	Catalog #	Antibody Incubation	Dilution
pTau	AT8	Mouse	Thermo Fisher	MN1020	4°C overnight	1:1000
ß-Amyloid	BAM-10	Mouse	Millipore	A3981	4°C overnight	1:1000
pTDP-43	-	Rabbit	Proteintech	22309-1-AP	4°C overnight	1:1000
TMEM106B[12]	-	-	-	-	4°C overnight	1:800
HRP Goat anti-Rabbit IgG	-	Goat	BD Biosciences	AB 395213	1 h at 21°C	1:8000
HRP Goat anti-Mouse IgG	-	Goat	Millipore	12-349	1 h at 21°C	1:8000

Immunofluorescence

Antibody	Clone	Species	Vendor	Catalog #	Antibody Incubation	Dilution

pTau	AT8	Mouse	Thermo Fisher	MN1020	4ºC overnight	1:200
MAP2	-	Chicken	Novus	NB300-213	4ºC overnight	1:200

References:

1. Adams PD, Afonine PV, Bunkóczi G, Chen VB, Davis IW, Echols N, Headd JJ, Hung L-W, Kapral GJ, Grosse-Kunstleve RW, McCoy AJ, Moriarty NW, Oeffner R, Read RJ, Richardson DC, Richardson JS, Terwilliger TC, Zwart PH (2010) PHENIX: a comprehensive Python-based system for macromolecular structure solution. Acta Cryst D 66:213–221. doi: 10.1107/S0907444909052925

2. Arseni D, Chen R, Murzin AG, Peak-Chew SY, Garringer HJ, Newell KL, Kametani F, Robinson AC, Vidal R, Ghetti B, Hasegawa M, Ryskeldi-Falcon B (2023) TDP-43 forms amyloid filaments with a distinct fold in type A FTLD-TDP. Nature 620:898–903. doi: 10.1038/s41586-023-06405-w

3. Attems J, Toledo JB, Walker L, Gelpi E, Gentleman S, Halliday G, Hortobagyi T, Jellinger K, Kovacs GG, Lee EB, Love S, McAleese KE, Nelson PT, Neumann M, Parkkinen L, Polvikoski T, Sikorska B, Smith C, Grinberg LT, Thal DR, Trojanowski JQ, McKeith IG (2021) Neuropathological consensus criteria for the evaluation of Lewy pathology in post-mortem brains: a multi-centre study. Acta Neuropathol 141:159–172. doi: 10.1007/s00401-020-02255-2

4. Braak H, Alafuzoff I, Arzberger T, Kretzschmar H, Del Tredici K (2006) Staging of Alzheimer disease-associated neurofibrillary pathology using paraffin sections and immunocytochemistry. Acta Neuropathol 112:389–404. doi: 10.1007/s00401-006-0127-z

5. Burt A, Toader B, Warshamanage R, von Kügelgen A, Pyle E, Zivanov J, Kimanius D, Bharat TAM, Scheres SHW (2024) An image processing pipeline for electron cryo-tomography in RELION-5. FEBS Open Bio 14:1788–1804. doi: 10.1002/2211-5463.13873

6. Emsley P, Lohkamp B, Scott WG, Cowtan K (2010) Features and development of Coot. Acta Cryst D 66:486–501. doi: 10.1107/S0907444910007493

7. Falcon B, Zhang W, Schweighauser M, Murzin AG, Vidal R, Garringer HJ, Ghetti B, Scheres SHW, Goedert M (2018) Tau filaments from multiple cases of sporadic and inherited Alzheimer's disease adopt a common fold. Acta Neuropathol 136:699–708. doi: 10.1007/s00401-018-1914-z

8. He S, Scheres SHW (2017) Helical reconstruction in RELION. Journal of Structural Biology 198:163–176. doi: 10.1016/j.jsb.2017.02.003

9. Jamali K, Käll L, Zhang R, Brown A, Kimanius D, Scheres SHW (2024) Automated model building and protein identification in cryo-EM maps. Nature 628:450–457. doi: 10.1038/s41586-024-07215-4

10. Lövestam S, Li D, Wagstaff JL, Kotecha A, Kimanius D, McLaughlin SH, Murzin AG, Freund SMV, Goedert M, Scheres SHW (2024) Disease-specific tau filaments assemble via polymorphic intermediates. Nature 625:119–125. doi: 10.1038/s41586-023-06788-w

11. Lövestam S, Scheres SHW (2022) High-throughput cryo-EM structure determination of amyloids. Faraday Discuss 240:243–260. doi: 10.1039/D2FD00034B

12. Marks JD, Ayuso VE, Carlomagno Y, Yue M, Todd TW, Hao Y, Li Z, McEachin ZT, Shantaraman A, Duong DM, Daughrity LM, Jansen-West K, Shao W, Calliari A, Bejarano

JG, DeTure M, Rawlinson B, Casey MC, Lilley MT, Donahue MH, Jawahar VM, Boeve BF, Petersen RC, Knopman DS, Oskarsson B, Graff-Radford NR, Wszolek ZK, Dickson DW, Josephs KA, Qi YA, Seyfried NT, Ward ME, Zhang Y-J, Prudencio M, Petrucelli L, Cook CN (2024) TMEM106B core deposition associates with TDP-43 pathology and is increased in risk SNP carriers for frontotemporal dementia. Science Translational Medicine 16:eadf9735. doi: 10.1126/scitranslmed.adf9735

13. Montine TJ, Phelps CH, Beach TG, Bigio EH, Cairns NJ, Dickson DW, Duyckaerts C, Frosch MP, Masliah E, Mirra SS, Nelson PT, Schneider JA, Thal DR, Trojanowski JQ, Vinters HV, Hyman BT (2012) National Institute on Aging–Alzheimer's Association guidelines for the neuropathologic assessment of Alzheimer's disease: a practical approach. Acta Neuropathol 123:1–11. doi: 10.1007/s00401-011-0910-3

14. Nelson PT, Lee EB, Cykowski MD, Alafuzoff I, Arfanakis K, Attems J, Brayne C, Corrada MM, Dugger BN, Flanagan ME, Ghetti B, Grinberg LT, Grossman M, Grothe MJ, Halliday GM, Hasegawa M, Hokkanen SRK, Hunter S, Jellinger K, Kawas CH, Keene CD, Kouri N, Kovacs GG, Leverenz JB, Latimer CS, Mackenzie IR, Mao Q, McAleese KE, Merrick R, Montine TJ, Murray ME, Myllykangas L, Nag S, Neltner JH, Newell KL, Rissman RA, Saito Y, Sajjadi SA, Schwetye KE, Teich AF, Thal DR, Tomé SO, Troncoso JC, Wang S-HJ, White CL, Wisniewski T, Yang H-S, Schneider JA, Dickson DW, Neumann M (2023) LATE-NC staging in routine neuropathologic diagnosis: an update. Acta Neuropathol 145:159–173. doi: 10.1007/s00401-022-02524-2

15. Pettersen EF, Goddard TD, Huang CC, Meng EC, Couch GS, Croll TI, Morris JH, Ferrin TE (2021) UCSF ChimeraX: Structure visualization for researchers, educators, and developers. Protein Science 30:70–82. doi: 10.1002/pro.3943

16. Rohou A, Grigorieff N (2015) CTFFIND4: Fast and accurate defocus estimation from electron micrographs. Journal of Structural Biology 192:216–221. doi: 10.1016/j.jsb.2015.08.008

17. Scheres SHW (2020) Amyloid structure determination in RELION-3.1. Acta Cryst D 76:94–101. doi: 10.1107/S2059798319016577

18. Schorb M, Haberbosch I, Hagen WJH, Schwab Y, Mastronarde DN (2019) Software tools for automated transmission electron microscopy. Nat Methods 16:471–477. doi: 10.1038/s41592-019-0396-9

19. Thal DR, Rüb U, Orantes M, Braak H (2002) Phases of Aβ-deposition in the human brain and its relevance for the development of AD. Neurology 58:1791–1800. doi: 10.1212/WNL.58.12.1791

20. Zivanov J, Nakane T, Scheres SHW (2019) A Bayesian approach to beam-induced motion correction in cryo-EM single-particle analysis. IUCrJ 6:5–17. doi: 10.1107/S205225251801463X
